# Molecular Dynamics Simulations of Deformable Viral Capsomers

**DOI:** 10.3390/v15081672

**Published:** 2023-07-31

**Authors:** Lauren B. Nilsson, Fanbo Sun, J. C. S. Kadupitiya, Vikram Jadhao

**Affiliations:** Intelligent Systems Engineering, Indiana University, Bloomington, IN 47408, USA; laurennilsson1@gmail.com (L.B.N.); fanbsun@iu.edu (F.S.); kadu@iu.edu (J.C.S.K.)

**Keywords:** viral capsids, self-assembly, elasticity, soft capsomers, coarse-grained models, molecular dynamics simulations, deformable nanostructures

## Abstract

Most coarse-grained models of individual capsomers associated with viruses employ rigid building blocks that do not exhibit shape adaptation during self-assembly. We develop a coarse-grained general model of viral capsomers that incorporates their stretching and bending energies while retaining many features of the rigid-body models, including an overall trapezoidal shape with attractive interaction sites embedded in the lateral walls to favor icosahedral capsid assembly. Molecular dynamics simulations of deformable capsomers reproduce the rich self-assembly behavior associated with a general T=1 icosahedral virus system in the absence of a genome. Transitions from non-assembled configurations to icosahedral capsids to kinetically-trapped malformed structures are observed as the steric attraction between capsomers is increased. An assembly diagram in the space of capsomer–capsomer steric attraction and capsomer deformability reveals that assembling capsomers of higher deformability into capsids requires increasingly large steric attraction between capsomers. Increasing capsomer deformability can reverse incorrect capsomer–capsomer binding, facilitating transitions from malformed structures to symmetric capsids; however, making capsomers too soft inhibits assembly and yields fluid-like structures.

## 1. Introduction

Icosahedral virus capsids [1] are nanoscale biological containers that are capable of spontaneously self-assembling from many copies of one or a few types of proteins [2]. This self-assembly can occur in the absence of a genome and can be reproduced by experiments in vitro [3]. In many cases, the basic assembly unit (“monomer”), also referred to as a capsomer or a subunit, is a small oligomer formed by a combination of a few proteins. The size of the capsomer depends on the virus. For example, the capsomer for minute virus of mice (MVM) capsids is a trimer (combination of three proteins) [4], and the capsomer for hepatitis B virus (HBV) capsids is a dimer (combination of two proteins) [5,6]. Experiments on evaluating the structure and assembly kinetics of virus capsids [7,8,9,10,11,12,13,14] have inspired many modeling and simulation efforts to understand the self-assembly process [15,16,17,18,19]. Due to large computational costs, simulations of capsid assembly starting from individual capsomers described with atomic resolution are presently limited to very short timescales or to specific elements of the assembly process [18]. Many computational studies trade off resolution for efficiency and employ coarse-graining to design simplified model representations of capsomers that effectively capture the key interactions between them [20,21,22,23,24,25,26,27,28,29,30]. The aim is often to construct a minimal model that elucidates the general organization principles governing the capsid self-assembly process.

A wide variety of coarse-grained models have been employed to realize the spontaneous formation of the symmetric icosahedral capsid shells starting from a mixture of dispersed model capsomers [15,16,17]. Molecular dynamics (MD) simulations of these models have been used to probe the link between the assembly products and the capsomer attributes, capsomer–capsomer interactions, and solution conditions. A large subset of these models describes the capsomer as a rigid structure built by fusing together a network of spheres (beads) into asymmetric triangular or trapezoidal-shaped blocks [17,20,24,26,31], which have been shown to favor the assembly into icosahedral capsids. In general, the capsid assembly is driven by highly directional short-range capsomer–capsomer attraction provided by the hydrophobic residues, which is realized in trapezoidal-shaped capsomers by embedding attractive interaction sites in the lateral sides of the capsomer [20,31]. The capsid assembly is sensitive to the strength of the attractive (binding) energy between the capsomers [15,26]—a weak binding energy may not overcome the entropic forces that favor a mixed configuration of dispersed capsomers, inhibiting capsid nucleation. And if the capsomer–capsomer binding energy is too strong, the higher degree of non-reversibility of bonded capsomers can inhibit the correction of defects induced in the intermediate structures, leading to the formation of kinetically-trapped (malformed) aggregates.

It has been recognized that MD simulations of rigid-body models with fixed capsomer shape may not be well suited to study the more exotic virus assembly phenomena, such as dimorphism [10,32], where proteins assemble to form two distinct icosahedral capsid morphologies (characterized with different *T* numbers) under similar solution conditions. Another phenomenon that presents challenges for rigid-body models is generalized structural polymorphism [22,33], where icosahedral viral capsids coexist with a variety of non-icosahedral yet ordered capsules. Earlier work on understanding the mechanisms driving polymorphism in viruses [22,34,35] endowed coarse-grained model capsomers with internal degrees of freedom to capture the structural flexibility and simulated them using the discontinuous molecular dynamics (DMD) method, which is a faster but coarser way to simulate assembly compared to traditional MD simulations. More recently, Hagan and co-workers performed dynamic Monte Carlo simulations of a coarse-grained model of HBV protein dimers, which were represented as flexible edges characterized with parameters informed by atomistic-resolution data [36]. Simulations linked the dimorphic HBV capsid assembly into T=3 and T=4 icosahedral structures to the relatively low value of the HBV bending modulus (≈$40\;k_{B}T$).

Studies of the self-assembly of empty capsids with a single icosahedral morphology starting from trapezoidal-shaped capsomers have mainly focused on using rigid-body models [20,24,26,31]. Much effort has been invested in designing rigid trapezoidal capsomers with the “right” inclination angle of the lateral sides to the surface normal in order to produce capsid assembly-competent intermediate structures at high yield in simulations. However, these capsomers are the result of a coarse graining process that ignores the flexibility of the real protein subunit and associated structural fluctuations, which may affect the attractive capsomer–capsomer energy and solution conditions associated with the capsid assembly [36,37,38]. Incorporating protein softness and associated elastic interactions explicitly in such coarse-grained models is computationally expensive and has been attempted in a few studies by augmenting the coarse-grained model with supportive elastic networks [37,38]. For example, in a recent study [38], elasticity was incorporated via a harmonic stretching potential between the beads and the resulting model was used to probe the co-assembly of capsomers and a polyion into a T=1 capsid via MD simulations.

In this paper, we develop a coarse-grained model of deformable capsomers that incorporates their stretching and bending energies and use it to study the self-assembly behavior of a general T=1 virus capsid system in the absence of a genome. Our aim is to develop a general model of a deformable capsomer building on previous coarse-grained rigid-body models [20,31] and retaining many of their key advantages, including an overall trapezoidal shape. Capsomer binding is driven by attractive interaction sites embedded in the lateral walls of the trapezoidal-shaped structure. The competition between the strength of the associated steric capsomer–capsomer attraction and capsomer deformability is investigated in detail using MD simulations accelerated by a hybrid OpenMP/MPI parallel computing technique coupled with a fast neighbor listing procedure. Simulations of identical deformable capsomers in an NVT ensemble at 298 K reproduce the rich equilibrium self-assembly behavior associated with a T=1 icosahedral virus system in the absence of a genome. Transitions from non-assembled configurations to icosahedral capsids to kinetically-trapped malformed structures are observed as the steric attraction between capsomers is increased.

An assembly diagram in the space of capsomer–capsomer steric attraction and capsomer deformability reveals that assembling capsomers of higher deformability into capsids requires increasingly large steric attraction between capsomers. Changing deformability of capsomers at a fixed steric attraction has a pronounced effect on virus self-assembly. Increasing capsomer deformability enhances the average edge and angle fluctuations, which reverse incorrect capsomer–capsomer binding and facilitate transitions from malformed structures to symmetric capsids. However, if the capsomers become too soft, capsid assembly is inhibited owing to the large structural fluctuations, and fluid-like structures are observed. The use of elasticity as a control knob to alter assembly behavior demonstrated in our work has broad implications in the design of reconfigurable systems based on soft materials, where there is a keen interest in understanding how self-assembly is affected by building blocks that can spontaneously reconfigure during assembly [39,40,41,42,43,44,45].

## 2. Models and Methods

### 2.1. Model System

**Figure 1:**
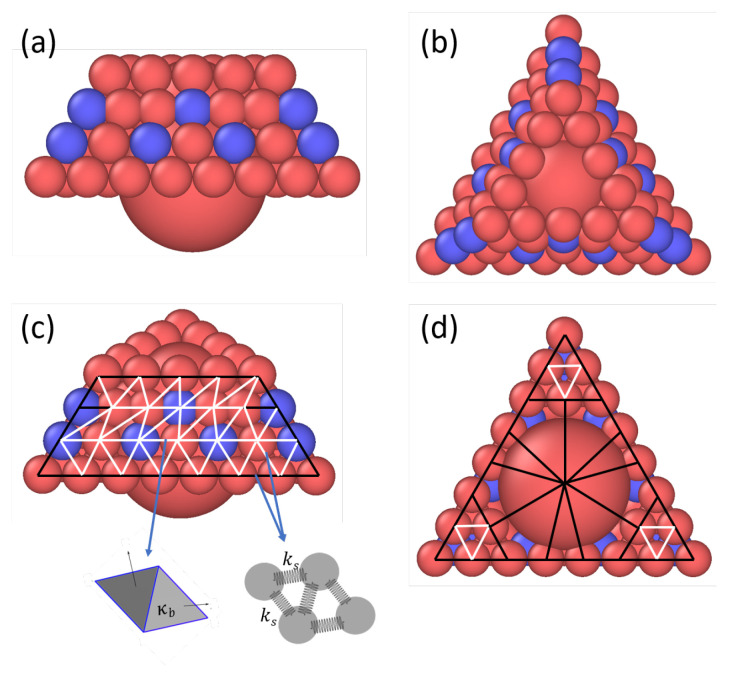
Side view (**a**) and top view (**b**) of the deformable capsomer. (**c**,**d**) show the perspective and bottom views, respectively, highlighting the edges associated with the bending and stretching of the capsomer. Blue beads attract blue beads on other capsomers, and red beads repel red and blue beads on other capsomers. White and black edges represent bending and non-bending edges, respectively. All bending edges are identical and characterized with a bending modulus $\kappa_{b}$. Edges between nearest-neighbor beads can stretch and are characterized with a spring constant $k_{s}$.

Following previous models aimed at studying the general aspects of the capsid self-assembly [31], the MVM protein is used as an example of a T=1 capsid system to aid the model capsomer design. Our coarse-grained deformable capsomer model is based on the rigid-body model developed by Mahalik and Muthukumar [31] built using the MVM trimers, which experiments [4] have shown are the key intermediates in the assembly of an MVM capsid. Figure 1 shows the side (a), top (b), perspective (c), and bottom (d) views of the deformable capsomer. The mass of each capsomer is taken to be that of an MVM trimer (≈2.5 kDa) and is distributed uniformly among its 78 spherical beads (each bead has a mass of ≈32 Da), and 76 out of 78 beads are identical in size and have a radius of 0.5 nm. These beads are arranged into four layers as shown in Figure 1a.

All layers are constructed as triangular frames with the inside of the layer being empty. The first (bottom) layer in Figure 1a is exposed to the exterior of the capsid and has an edge comprising 8 beads. The second layer with an edge comprising 7 beads sits on top of the indentations of the first layer. Similarly, the third layer with an edge comprising 7 beads is situated atop the indentations of the second layer; however, the beads in the third layer are placed closer together. The three-layered structure is capped by a fourth layer with an edge comprising 5 beads. This arrangement produces an asymmetric trapezoidal or truncated prism-shaped structure with an inclination angle of $53.9^{\circ }$ [4,31], as shown in Figure 1a. To reduce computational costs, instead of using a space-filling triangular lattice, the space enclosed within this structure is filled with one large bead of radius 1.8 nm placed in the middle of the first (bottom) layer and another large bead of radius 1.2 nm placed in the middle between the third and fourth (top) layers, as shown in Figure 1.

Steric attraction between capsomers is driven by the blue beads embedded in the second and third layers. Red beads of a capsomer interact via purely-repulsive steric forces with all the beads associated with a different capsomer. No steric interactions are considered between beads belonging to the same capsomer. The deformability of the capsomer is incorporated by connecting the beads with an elastic network represented by harmonic stretching and bending potentials, which enables the capsomer to stretch and bend. All nearest-neighbor beads are linked with harmonic springs characterized by identical spring constants. All edges in the middle of each lateral side except for the edges in the third layer connecting the outermost blue bead are bending edges (white), as shown in Figure 1. All bending edges are identical and characterized by the same bending modulus. All outermost edges and the edges connecting the larger beads to the smaller beads are taken to be non-bending edges (black). These choices are made in order to preserve the overall capsomer structural framework during self-assembly.

(1) $$H=\sum_{i=1}\sum_{j>1}4\varepsilon_{ij}\left[{\left(\frac{\sigma_{ij}}{r_{ij}}\right)}^{12}-{\left(\frac{\sigma_{ij}}{r_{ij}}\right)}^{6}\right]+\sum_{c=1}^{N}\sum_{e=1}^{E_{b}}\frac{\kappa_{b}}{2}{\left|{\hat{n}}_{c,e_{1}}-{\hat{n}}_{c,e_{2}}\right|}^{2}+\sum_{c=1}^{N}\sum_{e=1}^{E_{s}}\frac{k_{s}}{2}{\left(l_{c,e}-a_{e}\right)}^{2}$$

Equation (1) shows the Hamiltonian *H* describing a system of *N* deformable capsomers. The first term represents the sum of the inter-capsomer steric (excluded volume) interaction energies which are modeled via the truncated and shifted Lennard–Jones (LJ) potentials. Indices *i* and *j* sweep over beads in different capsomers, $r_{ij}$ is the distance between the beads, and $\sigma_{ij}$ represents the standard LJ bead size parameter. Purely repulsive interaction between beads is modeled by choosing the LJ well depth parameter $\varepsilon_{ij}=1k_{B}T$, the cutoff distance $r_{ij}^{cut}=2^{1/6}\sigma_{ij}$, and the associated energy shift $\varepsilon_{ij}^{\mathrm{shift}}=1k_{B}T$. Steric attraction between beads is modeled by choosing $\varepsilon_{ij}=\epsilon_{att}$, $r_{ij}^{cut}=2.5\sigma_{ij}$, and $\varepsilon_{ij}^{\mathrm{shift}}=4\epsilon_{att}\left({\left(\sigma_{ij}/r_{ij}^{cut}\right)}^{12}-{\left(\sigma_{ij}/r_{ij}^{cut}\right)}^{6}\right)$, where $\epsilon_{att}$ represents the strength of the steric attraction which governs the assembly of capsomers and serves as a tunable parameter in the model.

The second and third terms in Equation (1) are the total bending and stretching energy, respectively, and together encapsulate the total intra-capsomer energy associated with a system of deformable capsomers. Indices *c* and *e* sweep, respectively, over the number of capsomers *N* and the total number of bending edges $E_{b}$ in the second term and stretching edges $E_{s}$ in the third term. We introduce bending modulus $\kappa_{b}$ and stretching modulus (spring constant) $k_{s}$ as intrinsic attributes of a capsomer that effectively characterize the short-range elastic interactions. A bending penalty is applied to edges that separate planar triangular faces as shown in Figure 1. Bending energy associated with a bending edge *e* depends on the normals ${\hat{n}}_{c,e_{1}}$ and ${\hat{n}}_{c,e_{2}}$ to the adjoining faces and is measured relative to the planar conformation, which is assigned zero energy. Stretching energy associated with an edge of length $l_{c,e}$ is measured relative to the initial equilibrium edge length $a_{e}$ which depends on the edge *e*. The capsomer resistance to deformation is dependent on the values of the elastic moduli $\kappa_{b}$ and $k_{s}$, which serve as tunable parameters. Experiments and theoretical calculations have estimated that the virus bending modulus $\kappa_{b}$ and stretching modulus $k_{s}$ can vary by 2 orders of magnitude in the range of 10–1000 $k_{B}T$ and 10–1000 $k_{B}T/{\mathrm{nm}}^{2}$, respectively [33,46,47,48,49]. We are interested in probing the self-assembly behavior of capsomers over a wide range of deformabilities that includes values within the estimated range of virus elastic moduli. Accordingly, we associate rigid capsomers with elastic moduli $\left(\kappa_{b},k_{s}\right)=\left(5000,5000\right)$, and design four sets of deformable capsomers with increasing “softness”: $\left(\kappa_{b},k_{s}\right)=\left(10,1000\right),\;\left(10,500\right),\;\left(10,300\right),\;\left(5,300\right)$, where $\kappa_{b}$ values are in units of $k_{B}T$ and $k_{s}$ values are in units of $k_{B}T/{\mathrm{nm}}^{2}$.

A key focus of this paper is to explore how the competition between the capsomer–capsomer steric attraction and capsomer deformability changes assembly behavior. This competition is encapsulated in the minimal model system of capsomers described by the Hamiltonian *H*. We aim to determine the equilibrium assembly states of *H* as a function of the steric attraction $\epsilon_{att}$ and the elastic moduli $\kappa_{b}$ and $k_{s}$. We realize that other important capsomer features, such as capsomer charge, are ignored in this model. The model can be readily extended to include capsomer charge and the associated intra-capsomer and inter-capsomer electrostatic energies, as demonstrated in Section 3.5.

### 2.2. Molecular Dynamics Simulations

The time evolution of the model system described by the Hamiltonian *H* is performed using MD simulations with the goal to extract equilibrium assembly configurations and associated quantitative metrics. A reduced (dimensionless) system of units is adopted to express the model variables and simulation outputs: length is expressed in units of the small bead diameter (1 nm), mass is expressed in units of bead mass (32 Da), and energy is measured in units of $298k_{B}$ Joules. This set of base units leads to units of time, temperature, bending modulus, and stretching modulus as 3.5 picoseconds, 298 K, 298 $k_{B}$, and 298 $k_{B}/{\mathrm{nm}}^{2}$, respectively. In dimensionless units, the rigid capsomers correspond to bending modulus $\kappa_{b}=5000$ and stretching modulus $k_{s}=5000$, and the deformable capsomers are characterized with the following combinations of elastic moduli: $\left(\kappa_{b},k_{s}\right)=\left(10,1000\right),\;\left(10,500\right),\;\left(10,300\right),\;\left(5,300\right)$.

All simulations use 100 deformable capsomers in a cubic box of edge length L≈59 in reduced units (≈59 nm) with periodic boundary conditions. This setup corresponds to a protein concentration of 800 μM. Simulations are initialized by randomly placing the capsomers inside the box (avoiding overlaps) and by randomly drawing initial velocities of the associated beads from a uniform distribution between −0.5 and 0.5 such that the average bead velocity is 0. Simulations are performed in an NVT ensemble at a constant temperature of 298 K which is imposed using a Nosé–Hoover chain thermostat. Newton’s equations of motion are integrated using the velocity Verlet algorithm with a timestep of 0.002 (7 fs) and 0.0005 (1.8 fs) for simulations of rigid and deformable capsomers, respectively. The ≈4× smaller timestep for simulations of deformable capsomers compared to the rigid ones is to ensure energy conservation in the case of the former. All simulations are run for a total time of ≈200 ns, which entails simulating for 30 million steps for rigid capsomers and 120 million steps for deformable capsomers.

Simulations of 100 capsomers for millions of computational steps are enabled by using a hybrid OpenMP/MPI parallelization technique, which accelerates the force computations, and by implementing an efficient neighbor listing procedure. For the latter, each capsomer is treated as a mobile “cell”, and a Verlet-type [50] search over all capsomers is conducted when building the neighbor list. This is followed by a narrower search of beads within each capsomer. Overall, these enhancements result in a strong scaling of $𝒪(N_{b}\log N_{b})$ in the simulated number of beads $N_{b}$, enabling simulations to reach ≈10 million steps per day for deformable capsomers on 96 cores.

Desired quantities such as steric and elastic energies, temperature, and positions of beads are collected every 500,000 computational steps post equilibration. Quantitative metrics, such as maximum cluster size $N_{max}$, average cluster size $N_{av}$, and average edge length and angle fluctuations are extracted to assess the assembly behavior. $N_{max}$ and $N_{av}$ are defined using the size $N_{c}$ of the cluster formed by capsomers—a capsomer is considered to be a part of a cluster if its distance from at least one member of the cluster is less than a pre-selected cutoff distance $d_{cut}$, which we set to $d_{cut}=1.5\sigma$. In general, $N_{c}$ ranges from 1 to *N*, where *N* is the total number of capsomers in the simulation (in our case, $N_{c}$ ranges from 1 to 100). $N_{max}$ is defined as the maximum value of $N_{c}$ observed in the simulation and is computed as an ensemble average over a 2 ns time window. $N_{av}$ is defined for each $N_{c}$ as the average number of capsomers aggregated in a cluster of size $N_{c}$ over a 2 ns time window. In some cases, multiple replicas of simulations for the same model parameters are run in order to obtain converged statistical averages.

## 3. Results and Discussion

### 3.1. Rigid vs. Deformable Capsomers

**Figure 2:**
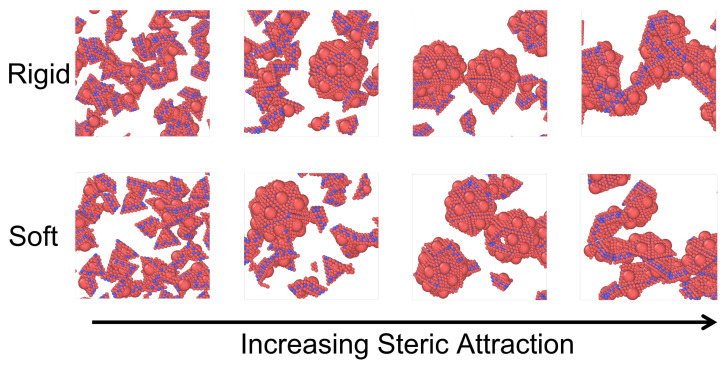
Steady-state configurations associated with the assembly of rigid capsomers characterized with elastic moduli $\kappa_{b}=5000$, $k_{s}=5000$ (top row) and deformable (“soft”) capsomers characterized with $\kappa_{b}=10$, $k_{s}=1000$ (bottom row) at 298 K as the steric attraction between capsomers is increased. In both cases, rich variations in the self-assembly behavior are observed (from left to right): non-assembled fluid, partial capsid assembly, nearly complete capsids, and malformed structures.

We begin by comparing the equilibrium states associated with the assembly of identical rigid capsomers and the assembly of identical deformable capsomers. Rigid capsomers are characterized with elastic moduli $\kappa_{b}=5000$, $k_{s}=5000$. As an example, we choose the deformable capsomers of elastic moduli $\kappa_{b}=10$, $k_{s}=1000$ to represent the “soft” system (Figure 2); the following discussion is similar for systems of deformable capsomers characterized with elastic moduli ($\kappa_{b},k_{s})=\left(10,500\right),\left(10,300\right),\left(5,300\right)$. In both cases, simulations of capsomers correspond to a protein concentration of 800 μM and are performed in an NVT ensemble at temperature T=298 K. Many in vitro experiments exploring capsid self-assembly behavior employ protein concentrations in the 1–100 μM range [12]. Our choice of a relatively high protein concentration is because of computational convenience as this yields a high volume packing fraction of proteins which “speeds up” the assembly process. Figure 2 top and bottom rows illustrate the representative snapshots associated with the assembly products obtained using simulations of rigid and deformable capsomers respectively. Similar to the results of previous studies [31,38], the rigid capsomer model produces a diversity of assembly products associated with a T=1 icosahedral virus system as the steric attraction between the capsomers is enhanced. For a relatively weak attraction between the capsomers, non-assembled fluid-like states are observed (leftmost snapshot). Increasing the strength of this attraction leads to partial capsid assembly or nearly-complete icosahedral capsids (middle two snapshots). However, if the steric attraction is too strong, the capsomers aggregate into kinetically trapped malformed structures (rightmost snapshot).

The bottom row shows that deformable capsomers can reproduce this rich self-assembly behavior with similar transitions from fluid to nearly-complete icosahedral capsids to kinetically-trapped malformed structures as the steric attraction between capsomers is increased. These transitions demonstrate the utility of a deformable capsomer model to investigate the assembly of protein subunits. As noted above, the timestep associated with simulations of deformable capsomers is ≈4× smaller compared to the ones with rigid capsomers. Thus, the steady-state associated with an assembly product takes 4× longer to reach in simulations of deformable capsomers compared to rigid capsomers.

### 3.2. Assembly Diagram

**Figure 3:**
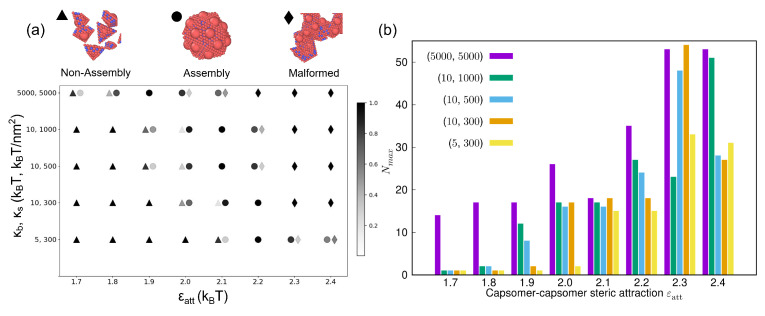
(**a**) A diagram showing the assembly products for the capsomers characterized with capsomer–capsomer steric attraction $\epsilon_{att}$ and capsomer deformability $\left(\kappa_{b},k_{s}\right)$. Legend at the top shows the assembly products: non-assembled fluid (triangles), nearly-complete capsid assembly (circles), and malformed structures (diamonds). The grayscale denotes the relative proportion of these products at a given statepoint ($\epsilon_{att},\kappa_{b},k_{s}$). Assembling capsomers of higher deformability into capsids requires increasingly large steric attraction. (**b**) Maximum cluster size $N_{max}$ vs. $\epsilon_{att}$ for capsomers of different elastic moduli $\left(\kappa_{b},k_{s}\right)$ noted in the legend.

We now conduct a systematic study of the link between the assembly behavior and the competition between the steric attraction and elastic interactions. Specifically, we examine the changes in the assembly behavior as a function of tuning the capsomer–capsomer steric attraction ($\epsilon_{att}$) and capsomer deformability ($\kappa_{b},k_{s}$). Forty model systems are designed via the combination of eight values of $\epsilon_{att}=1.7,1.8,1.9,2.0,2.1,2.2,2.3,2.4$, and five pairs of elastic moduli: ($\kappa_{b},k_{s})=\left(5000,5000\right),\left(10,1000\right),\left(10,500\right),\left(10,300\right),\left(5,300\right)$. Figure 3a presents the assembly diagram predicted by MD simulations for these 40 model systems. The capsomer assembly configurations can be classified into three broad types: a non-assembled fluid-like state with cluster size $N_{c}\leq 3$ (triangles), nearly-complete capsids with $N_{c}\geq 16$ (circles), and malformed structures (diamonds) with $N_{c}>20$. Many systems are best represented as a mixture of two of these assembly states [38]. For example, at $\epsilon_{att}=2$ and $\left(\kappa_{b},k_{s}\right)=\left(10,500\right)$, both non-assembled fluid-like state and nearly-complete capsids are observed. The relative proportions of these states is represented by the symbol color coded as a grayscale, which denotes $N_{av}/N$, i.e., the average number of capsomers $N_{av}$ of a cluster size $N_{c}$ normalized by the total number of capsomers *N*. The darker the symbol, the higher the fraction of the associated assembly product.

A common trend is observed for capsomers of different deformabilities: the capsomers transition from a non-assembled configuration to nearly-complete capsids to malformed structures with increasing steric attraction between capsomers. Figure 3a also shows that assembling capsomers of higher deformability into capsids requires increasingly large steric attraction between capsomers. This can be attributed to the higher entropic costs associated with softer capsomers compared to the rigid ones, which can only be overcome by a larger steric attraction between capsomers. The observed assembly transitions are quantified in Figure 3b via the maximum cluster size $N_{max}$ associated with the assembly state. Consistent with the assembly diagram, changes in $N_{max}$ show a transition for deformable capsomers of different elastic moduli from fluid-like states ($N_{max}\approx 1$) to symmetric capsids ($N_{max}\approx 20$) to malformed structures ($N_{max}\approx 50$) with increasing $\epsilon_{att}$.

### 3.3. Effects of Changing Steric Attraction

**Figure 4:**
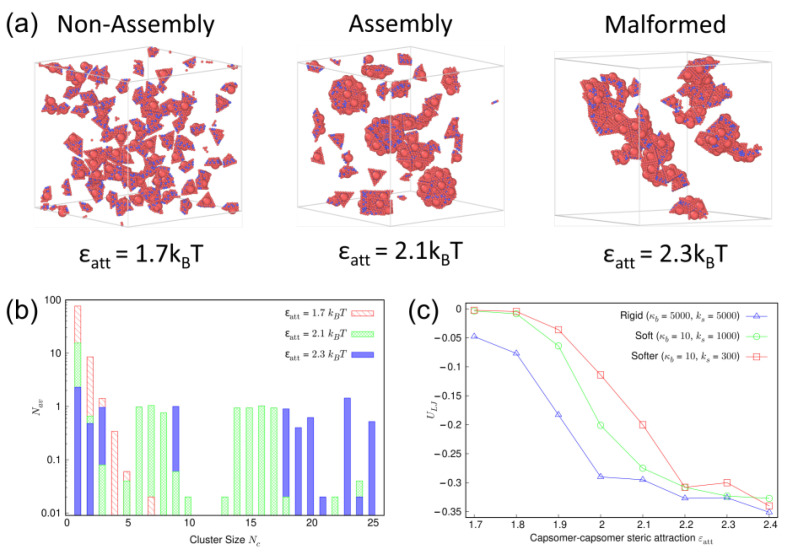
(**a**) Representative simulation snapshots of fluid-like configuration, capsid assembly, and malformed structures observed for a system of deformable capsomers at the indicated steric attraction values. (**b**) Average number $N_{av}$ of capsomers aggregated in a cluster of size $N_{c}$ associated with each of the three assembly states shown in (**a**). (**c**) Steric energy per bead, $U_{LJ}$, vs. steric attraction between capsomers for different elastic moduli ($\kappa_{b}$ in units of $k_{B}T$ and $k_{s}$ in units of $k_{B}T/{\mathrm{nm}}^{2}$).

We now closely examine the assembly transitions observed for the deformable capsomers with increasing steric attraction parameter $\epsilon_{att}$. For the sake of illustration, we consider the case of capsomers with elastic moduli $\left(\kappa_{b},k_{s}\right)=\left(10,1000\right)$. Figure 4a shows the representative simulation snapshots of the three distinct assembly states: fluid-like, capsid assembly, and malformed structures observed for this deformable capsomer system at $\epsilon_{att}=1.7,2.1,2.3$, respectively. Average number $N_{av}$ of capsomers aggregated in a cluster of size $N_{c}$ associated with each of these states are shown in Figure 4b. Recall that $N_{av}$ lies between 0 and the total number N=100 of capsomers. At a weak steric attraction $\epsilon_{att}=1.7$ (red striped bars), capsomers generally remain separated from each other and a large number ($N_{av}\approx 90$) of single capsomers of cluster size $N_{c}=1$ is recorded. Aggregates formed by two capsomers and three capsomers are also observed. $N_{av}$ drops sharply with increasing $N_{c}$, with almost no aggregates ($N_{av}<0.1$) observed for $N_{c}>5$. These signatures are consistent with a fluid-like system [51].

For the nearly complete capsid assembly obtained at $\epsilon_{att}=2.1$ (green cross-pattern bars), the number of single capsomers is significantly decreased to $N_{av}\approx 10$. This reduction and similar reduction in the two-capsomer and three-capsomer aggregates is traded off with an increase in $N_{av}$ associated with on-pathway intermediates characterized with cluster sizes $N_{c}\in \left(14,18\right)$. Further increase in the steric attraction to $\epsilon_{att}=2.3$ (blue solid bars) yields higher values of $N_{av}$ for cluster sizes $N_{c}≳20$. These large aggregates are associated with malformed structures. Distributions of $N_{av}$ similar to the ones shown in Figure 4b are observed for capsomers of other deformabilities, albeit at different steric attraction values.

Figure 4c shows the variation in the steric energy $U_{LJ}$ per bead associated with these assembly states (green circles) with increasing steric attraction $\epsilon_{att}$. Steric energy exhibits a sudden drop in the range $\epsilon_{att}\in \left(1.8,2.1\right)$, which can be linked to the transition from fluid-like structure to ordered capsid assembly. Similar drops in the steric energy are also observed for the rigid (blue triangles) and the softer capsomer (red squares) systems although at different steric attraction values. For example, in the rigid case the drop occurs for comparatively smaller $\epsilon_{att}$. All three systems exhibit a similar shape for the variation of the steric energy vs. $\epsilon_{att}$, but as the capsomers become softer, the trend is right-shifted, which is consistent with the observation that higher steric attraction is required to assemble increasingly soft capsomers into capsids.

### 3.4. Effects of Tuning Capsomer Deformability

**Figure 5:**
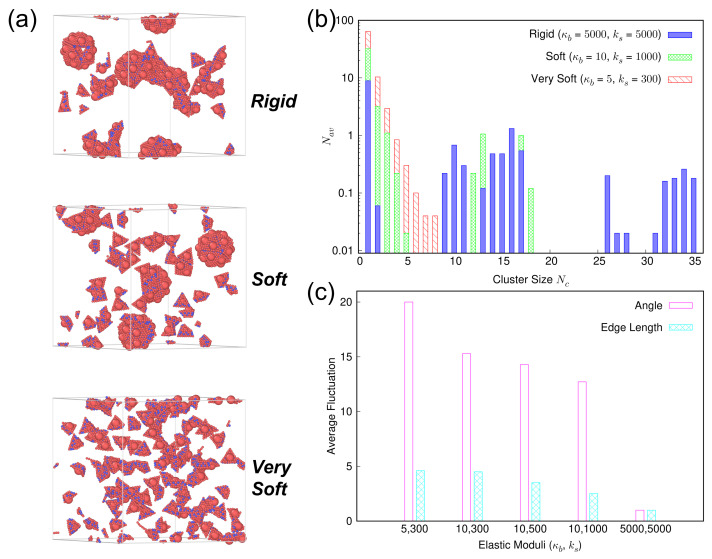
(**a**) Representative snapshots of the assembly states formed by rigid, soft, and very soft capsomers characterized with elastic moduli $\left(\kappa_{b},k_{s}\right)=\left(5000,5000\right)$, (10,1000), and (5,300), respectively under the same steric attraction $\epsilon_{att}=2$, where $\kappa_{b}$ is the bending modulus (in $k_{B}T$) and $k_{s}$ is the stretching modulus (in $k_{B}T/{\mathrm{nm}}^{2}$). (**b**) Average number $N_{av}$ of capsomers aggregated in a cluster of size $N_{c}$ associated with capsomers characterized with different $\left(\kappa_{b},k_{s}\right)$. (**c**) Average angle fluctuations (magenta clear bars) and edge length fluctuations (cyan filled bars) vs. capsomer elastic moduli. Fluctuations are normalized by their respective values for the rigid case characterized with $\left(\kappa_{b},k_{s}\right)=\left(5000,5000\right)$. Structural fluctuations increase with increasing capsomer deformability.

Endowing the capsomers with the potential to bend and stretch unlocks the possibility of controlling the assembly behavior by tuning the capsomer deformability alone, while keeping the inter-capsomer interactions the same. Figure 5a illustrates this scenario via three representative simulation snapshots of the assembly states formed by capsomers of different deformabilities under the same steric attraction $\epsilon_{att}=2$. For the rigid case (top) characterized with $\left(\kappa_{b},k_{s}\right)=\left(5000,5000\right)$, a mixture of malformed structures and partially-complete capsids is observed. As the rigidity of the capsomer is decreased to $\left(\kappa_{b},k_{s}\right)=\left(10,1000\right)$, which we term the “soft” case (middle), the assembly state transforms into nearly-complete capsids. One explanation for this transformation is that higher deformability increases the entropic gain associated with reversing incorrect capsomer–capsomer binding, which can facilitate the formation of on-pathway-competent intermediates. However, if the capsomers become too soft, as exemplified by the system characterized with $\left(\kappa_{b},k_{s}\right)=\left(5,300\right)$, a non-assembled fluid is obtained (bottom). The steric attraction parameter $\epsilon_{att}=2$ results in an average total binding energy per capsomer face of ≈14 $k_{B}T$. The transformation from an ordered structure to a fluid-like configuration at a fixed steric attraction strength can be attributed to this binding energy being insufficient to overcome the much greater entropic costs associated with these very soft capsomers. The high entropy enables the capsomers to lower the net free energy by remaining separated instead of aggregating.

Figure 5b shows the average number $N_{av}$ of capsomers aggregated in a cluster of size $N_{c}$ associated with these three distinct assembly behaviors. The results are consistent with the observed transition in Figure 5a. Significant $N_{av}$ values for large cluster sizes $N_{c}\in \left(25,35\right)$ are observed for rigid capsomers (blue solid bars), signaling the presence of malformed structures. On the contrary, no such large-sized aggregates are observed for the “soft” case (green cross-pattern bars). Instead, larger $N_{av}$ values are recorded for the single capsomers and few-capsomer aggregates ($N_{c}\in \left(1,5\right)$), which signals the reversing of the incorrectly bonded capsomers. Further, $N_{av}$ values are also large for cluster sizes $N_{c}\in \left(11,18\right)$ associated with partially and nearly-complete capsid assembly. Finally, for the “too soft” case (red striped bars), the bulk of the capsomers are present either as single capsomers or few-capsomer aggregates with cluster sizes $N_{c}\in \left(1,5\right)$.

To gain further insight into the pronounced effect of changing deformability of capsomers on the assembly behavior at a fixed capsomer–capsomer steric attraction, we extract the average angle and edge length fluctuations for capsomers of different elastic moduli. Figure 5c reports these fluctuations normalized by their respective values for the rigid case $\left(\kappa_{b},k_{s}\right)=\left(5000,5000\right)$. Both types of structural fluctuations increase as the deformability of the capsomers is enhanced. An increase of edge length fluctuations by ≈2× and angle fluctuations by ≈12× for the deformable capsomer system characterized with $\left(\kappa_{b},k_{s}\right)=\left(10,1000\right)$ provides the entropic freedom for capsomers to reverse incorrect capsomer–capsomer binding and redirect binding that facilitates transitions from malformed structures to on-pathway-competent intermediates. However, for capsomers that are too soft, e.g., the ones characterized with $\left(\kappa_{b},k_{s}\right)=\left(5,300\right)$, large increases in edge length fluctuations (≈5×) and angle fluctuations (≈20×) create a much greater entropic advantage for the capsomers to remain free in the solution. The available binding energy is unable to offset the entropic gain resulting from these structural fluctuations, which inhibits capsid assembly and favors fluid-like configurations.

### 3.5. Effects of Capsomer Surface Charge

In many cases, the proteins building up a virus capsid are charged. The presence of these surface charges can influence the competition between the steric attraction and the intra-capsomer elastic forces, thus altering the assembly behavior. We now present the results of a preliminary investigation that demonstrates the extension of the deformable capsomer model to charged capsomers. Following earlier models [31], charges are introduced in the fourth (top) layer of the trapezoid-shaped capsomer (see Figure 1b) by assigning a charge of 0.5 e to each of the three central beads associated with the three edges, which results in a total positive charge of 4.5 e for each capsomer. We consider the capsomers to be present in an aqueous solvent that is modeled implicitly via a dielectric permittivity $\epsilon_{d}$. The solvent inhabits a monovalent electrolyte of concentration *c* which is also modeled implicitly and has the effect of screening the Coulomb interactions between the beads.

The beads belonging to the same capsomer or to two different capsomers interact via screened electrostatic (Yukawa) potential energies, which are added to the intra-capsomer and the inter-capsomer components of the Hamiltonian shown in Equation (1). The electrostatic energy between the *i*th bead of charge $q_{i}$ and *j*th bead of charge $q_{j}$ at distance $r_{ij}$ is given by $l_{B}q_{i}q_{j}e^{-\kappa r_{ij}}/r_{ij}$, where $l_{B}\approx 0.7$ nm is the Bjerrum length of water and $\kappa =1/\lambda_{D}=\sqrt{8\pi l_{B}c}$ is the inverse of the screening (Debye) length $\lambda_{D}$. To speedup calculations via the neighbor listing procedure, the Yukawa potential is cut off after $12\lambda_{D}$.

**Figure 6:**
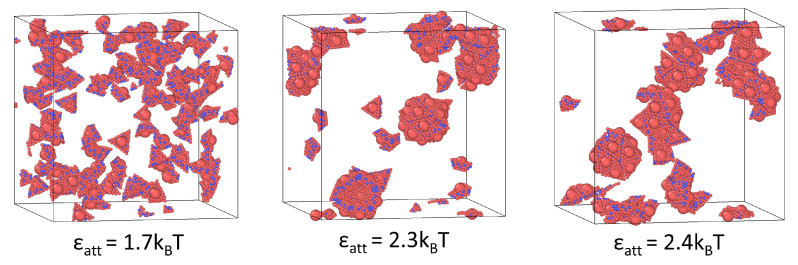
Assembly states of charged deformable capsomers characterized with $\left(\kappa_{b},k_{s}\right)=\left(10,300\right)$ for indicated values of steric attraction $\epsilon_{att}$. Non-assembled fluid, nearly-complete capsids and malformed structures form at $\epsilon_{att}=1.7,2.3,2.4$, respectively.

Figure 6 shows the assembly transitions of charged deformable capsomers characterized with $\left(\kappa_{b},k_{s}\right)=\left(10,300\right)$. Similar to the uncharged case, transitions from the fluid-like configuration to nearly-complete capsids to malformed structures are observed with increasing steric attraction $\epsilon_{att}$. The transitions occur at different values of $\epsilon_{att}=1.7$ (fluid), 2.3 (capsids), and 2.4 (malformed structures) compared to the uncharged case. For example, the uncharged system at $\epsilon_{att}=2.3$ exhibits a malformed structure (Figure 3a); however, when the surface charges are considered, this same system yields nearly-complete capsids. We attribute this shift to the repulsive electrostatic forces neutralizing part of the strong steric attraction at $\epsilon_{att}=2.3$, yielding a net binding energy that favors capsid assembly. In other words, the steric attraction resulting from the hydrophobic residues is attenuated by the repulsive electrostatic forces emerging from the charges present on the capsomers.

## 4. Conclusions

We have developed a coarse-grained general model of viral capsomers that incorporates their stretching and bending energies. MD simulations of identical deformable capsomers in an NVT ensemble at 298 K show that the combination of orientation-dependent capsomer–capsomer steric interactions and capsomer deformability can yield capsid structures with T=1 icosahedral symmetry as low-energy configurations. For a wide range of elastic moduli, transitions from non-assembled configurations to icosahedral capsids to kinetically-trapped malformed structures are observed as the steric attraction between capsomers is increased. Assembling capsomers of higher deformability into capsids requires increasingly large steric attraction between capsomers. In general, we expect the solution conditions yielding ordered capsids to be different for deformable capsomers compared to the rigid ones. The demonstration of deformable nanostructures self-assembling into ordered materials with appropriate adjustment of solution conditions has broad implications in using deformability as a control knob to change the self-assembly of soft materials.

Capsomer deformability has a pronounced effect on viral self-assembly. Deformability enables capsid assembly where there was none observed for rigid capsomers by enhancing the average angle and edge length fluctuations which reverse incorrect capsomer–capsomer binding and facilitate transitions from malformed structures to symmetric capsids. The lowering of elastic moduli can be considered as promoting optimal transient bond formation, i.e., a similar frequency of bond-breaking and bond-forming events, which has been linked to the effective self-assembly of ordered capsids [15]. However, making capsomers too soft produces large structural fluctuations which greatly enhance the probability of bond-breaking events, limiting the potential of capsid nucleation.

In this work, we tuned the deformability by changing both bending and stretching moduli. Our future work will systematically explore the effects of tuning one of these elastic parameters on the assembly states. Further, we will investigate the assembly of deformable capsomers around cargo such as nanoparticles or polyions [38]. Extending the deformable capsomer model to probe the link between capsomer deformability and the disassembly of viruses [52,53] will also be a subject of future work.

## Data Availability

The code, input data files, and example output datasets are publicly available on GitHub at https://github.com/softmaterialslab/capsid-souffle/, accessed on 30 July 2023.

## Abbreviations

The following abbreviations are used in this manuscript:

MVM	Minute virus of mice
HBV	Hepatitis B virus
MD	Molecular dynamics
LJ	Lennard–Jones

## References

[1] Caspar D.L., Klug A. (1962). Physical principles in the construction of regular viruses. Cold Spring Harbor Symposia on Quantitative Biology.

[2] Fraenkel-Conrat H., Williams R.C. (1955). Reconstitution of active tobacco mosaic virus from its inactive protein and nucleic acid components. Proc. Natl. Acad. Sci. USA.

[3] Sun S., Rao V.B., Rossmann M.G. (2010). Genome packaging in viruses. Curr. Opin. Struct. Biol..

[4] Reguera J., Carreira A., Riolobos L., Almendral J.M., Mateu M.G. (2004). Role of interfacial amino acid residues in assembly, stability, and conformation of a spherical virus capsid. Proc. Natl. Acad. Sci. USA.

[5] Zhou S., Standring D.N. (1992). Hepatitis B virus capsid particles are assembled from core-protein dimer precursors. Proc. Natl. Acad. Sci. USA.

[6] Conway J.F., Cheng N., Zlotnick A., Wingfield P.T., Stahl S.J., Steven A.C. (1997). Visualization of a 4-helix bundle in the hepatitis B virus capsid by cryo-electron microscopy. Nature.

[7] Prevelige P.E., Thomas D., King J. (1993). Nucleation and growth phases in the polymerization of coat and scaffolding subunits into icosahedral procapsid shells. Biophys. J..

[8] Zlotnick A., Aldrich R., Johnson J.M., Ceres P., Young M.J. (2000). Mechanism of capsid assembly for an icosahedral plant virus. Virology.

[9] Ceres P., Zlotnick A. (2002). Weak Protein-Protein Interactions Are Sufficient To Drive Assembly of Hepatitis B Virus Capsids. Biochemistry.

[10] Uetrecht C., Versluis C., Watts N.R., Roos W.H., Wuite G.J., Wingfield P.T., Steven A.C., Heck A.J. (2008). High-resolution mass spectrometry of viral assemblies: Molecular composition and stability of dimorphic hepatitis B virus capsids. Proc. Natl. Acad. Sci. USA.

[11] Chen C., Kao C.C., Dragnea B. (2008). Self-assembly of brome mosaic virus capsids: Insights from shorter time-scale experiments. J. Phys. Chem. A.

[12] Pierson E.E., Keifer D.Z., Selzer L., Lee L.S., Contino N.C., Wang J.C.Y., Zlotnick A., Jarrold M.F. (2014). Detection of Late Intermediates in Virus Capsid Assembly by Charge Detection Mass Spectrometry. J. Am. Chem. Soc..

[13] Harms Z.D., Selzer L., Zlotnick A., Jacobson S.C. (2015). Monitoring Assembly of Virus Capsids with Nanofluidic Devices. ACS Nano.

[14] Kondylis P., Zhou J., Harms Z.D., Kneller A.R., Lee L.S., Zlotnick A., Jacobson S.C. (2017). Nanofluidic Devices with 8 Pores in Series for Real-Time, Resistive-Pulse Analysis of Hepatitis B Virus Capsid Assembly. Anal. Chem..

[15] Hagan M.F. (2014). Modeling viral capsid assembly. Adv. Chem. Phys..

[16] Hagan M.F., Zandi R. (2016). Recent advances in coarse-grained modeling of virus assembly. Curr. Opin. Virol..

[17] Rapaport D. (2018). Molecular dynamics study of T = 3 capsid assembly. J. Biol. Phys..

[18] Hadden J.A., Perilla J.R. (2018). All-atom virus simulations. Curr. Opin. Virol..

[19] Lynch D.L., Pavlova A., Fan Z., Gumbart J.C. (2023). Understanding Virus Structure and Dynamics through Molecular Simulations. J. Chem. Theory Comput..

[20] Rapaport D. (2004). Self-assembly of polyhedral shells: A molecular dynamics study. Phys. Rev. E.

[21] Hagan M.F., Chandler D. (2006). Dynamic pathways for viral capsid assembly. Biophys. J..

[22] Nguyen H.D., Reddy V.S., Brooks C.L. (2007). Deciphering the kinetic mechanism of spontaneous self-assembly of icosahedral capsids. Nano Lett..

[23] Elrad O.M., Hagan M.F. (2008). Mechanisms of size control and polymorphism in viral capsid assembly. Nano Lett..

[24] Rapaport D. (2010). Modeling capsid self-assembly: Design and analysis. Phys. Biol..

[25] Elrad O.M., Hagan M.F. (2010). Encapsulation of a polymer by an icosahedral virus. Phys. Biol..

[26] Rapaport D. (2012). Molecular dynamics simulation of reversibly self-assembling shells in solution using trapezoidal particles. Phys. Rev. E.

[27] Zhang R., Linse P. (2013). Icosahedral capsid formation by capsomers and short polyions. J. Chem. Phys..

[28] Perlmutter J.D., Perkett M.R., Hagan M.F. (2014). Pathways for virus assembly around nucleic acids. J. Mol. Biol..

[29] Perlmutter J.D., Mohajerani F., Hagan M.F. (2016). Many-molecule encapsulation by an icosahedral shell. eLife.

[30] Wołek K., Cieplak M. (2017). Self-assembly of model proteins into virus capsids. J. Phys. Condens. Matter.

[31] Mahalik J., Muthukumar M. (2012). Langevin dynamics simulation of polymer-assisted virus-like assembly. J. Chem. Phys..

[32] Crowther R., Kiselev N., Böttcher B., Berriman J., Borisova G., Ose V., Pumpens P. (1994). Three-dimensional structure of hepatitis B virus core particles determined by electron cryomicroscopy. Cell.

[33] Moerman P., van der Schoot P., Kegel W. (2016). Kinetics versus Thermodynamics in Virus Capsid Polymorphism. J. Phys. Chem. B.

[34] Nguyen H.D., Brooks C.L. (2008). Generalized structural polymorphism in self-assembled viral particles. Nano Lett..

[35] Nguyen H.D., Reddy V.S., Brooks C.L. (2009). Invariant polymorphism in virus capsid assembly. J. Am. Chem. Soc..

[36] Mohajerani F., Tyukodi B., Schlicksup C.J., Hadden-Perilla J.A., Zlotnick A., Hagan M.F. (2022). Multiscale Modeling of Hepatitis B Virus Capsid Assembly and Its Dimorphism. ACS Nano.

[37] Globisch C., Krishnamani V., Deserno M., Peter C. (2013). Optimization of an elastic network augmented coarse grained model to study CCMV capsid deformation. PLoS ONE.

[38] Angelescu D.G. (2017). Assembled viral-like nanoparticles from elastic capsomers and polyion. J. Chem. Phys..

[39] Batista V.M., Miller M.A. (2010). Crystallization of deformable spherical colloids. Phys. Rev. Lett..

[40] Nguyen T.D., Jankowski E., Glotzer S.C. (2011). Self-assembly and reconfigurability of shape-shifting particles. ACS Nano.

[41] Zhang Y., Lu F., Van Der Lelie D., Gang O. (2011). Continuous phase transformation in nanocube assemblies. Phys. Rev. Lett..

[42] Brunk N.E., Jadhao V. (2019). Computational studies of shape control of charged deformable nanocontainers. J. Mater. Chem. B.

[43] Brunk N.E., Kadupitiya J., Jadhao V. (2020). Designing Surface Charge Patterns for Shape Control of Deformable Nanoparticles. Phys. Rev. Lett..

[44] Tanjeem N., Hall D.M., Minnis M.B., Hayward R.C., Grason G.M. (2022). Focusing frustration for self-limiting assembly of flexible, curved particles. Phys. Rev. Res..

[45] Manning M.L. (2023). Essay: Collections of Deformable Particles Present Exciting Challenges for Soft Matter and Biological Physics. Phys. Rev. Lett..

[46] May E.R., Brooks C.L. (2011). Determination of viral capsid elastic properties from equilibrium thermal fluctuations. Phys. Rev. Lett..

[47] May E.R., Aggarwal A., Klug W.S., Brooks C.L. (2011). Viral capsid equilibrium dynamics reveals nonuniform elastic properties. Biophys. J..

[48] Roos W., Bruinsma R., Wuite G. (2010). Physical virology. Nat. Phys..

[49] Carrasco C., Castellanos M., de Pablo P.J., Mateu M.G. (2008). Manipulation of the mechanical properties of a virus by protein engineering. Proc. Natl. Acad. Sci. USA.

[50] Verlet L. (1967). Computer “experiments” on classical fluids. I. Thermodynamical properties of Lennard-Jones molecules. Phys. Rev..

[51] Frenkel D., Smit B. (2001). Understanding Molecular Simulation: From Algorithms to Applications.

[52] Zandi R., Reguera D. (2005). Mechanical properties of viral capsids. Phys. Rev. E.

[53] Timmermans S.B., Ramezani A., Montalvo T., Nguyen M., van der Schoot P., van Hest J.C., Zandi R. (2022). The dynamics of viruslike capsid assembly and disassembly. J. Am. Chem. Soc..

